# Solidification cracking of laser melted commercial-purity tungsten

**DOI:** 10.1038/s41598-025-24742-w

**Published:** 2025-11-28

**Authors:** Venkata Satya Surya Amaranth Karra, Nicholas Lamprinakos, Yunhui Chen, Gabe Guss, Alexander Rack, Steven Van Petegem, Thejaswi U. Tumkur, Anthony D. Rollett, Petrus Christiaan Pistorius, Bryan A. Webler

**Affiliations:** 1 Department of Materials Science and Engineering, Carnegie Mellon University, Pittsburgh, PA 15213 USA; 2 Lawrence Livermore National Laboratory, 7000 East Ave, Livermore, 94550 USA; 3 RMIT Centre for Additive Manufacturing, RMIT University, Melbourne, 3000 Australia; 4 ESRF - The European Synchrotron Radiation Facility, 71 Avenue Des Martyrs, CS40220, 38043 Grenoble Cedex 9, France; 5 Center for Photon Science, Paul Scherrer Institut, Forschungsstr. 111, 5232 Villigen, Switzerland

**Keywords:** Engineering, Materials science

## Abstract

The high melting temperature of tungsten (W) makes it an attractive candidate for energy generation applications; however, its use is limited by its poor ductility at low temperatures. This limitation affects even melt-based additive manufacturing (AM) processes such as laser powder bed fusion (PBF-LB), with as-fabricated pure W exhibiting both longitudinal and transverse cracks. Metallurgical and processing factors that affect these cracks are still being explored. This work utilizes powderless single-tracks on pure W plates made over a range of power and velocity combinations in three unique PBF-LB setups: one with flowing argon, one with continuous vacuum, and an in situ high-speed synchrotron X-ray radiography setup. Each had different oxygen activities in their build environments. Longitudinal cracks with oxides appearing to exude from the crack were found for tracks deposited in a build environment with argon shield gas, while no such cracks were observed for builds conducted in vacuum. A calculation showed that direct oxygen ingress into the melt pool from the build environment is negligible when argon shield gas is present. Thus, incorporation of spatter is a likely mechanism for oxygen ingress into the melt pool. Radiography showed extensive keyholing, spatter generation, and crack formation at keyhole porosity. A heat transfer calculation showed the crack formation time was consistent with cooling below its ductile-to-brittle transition (DBT) temperature. This work identifies solidification cracking as a feasible mechanism in pure W beyond the well-known DBT related cracking.

**Supplementary Information:**

The online version contains supplementary material available at 10.1038/s41598-025-24742-w.

## Introduction

Compared with other materials, tungsten (W) combines a high melting point, good plasma radiation resistance, high thermal conductivity, and low thermal expansion coefficient, making it a possible candidate for applications at operating temperatures where superalloys fail. Tungsten, like many other body-centered cubic (BCC) metals, exhibits a ductile-to-brittle transition (DBT) temperature. One of the major challenges of fabricating parts from tungsten is that its DBT temperature is relatively high, between 200 and 500 °C, making tungsten extremely susceptible to cracking during cooling^1^.

While traditional fabrication techniques like powder metallurgy (PM), spark plasma sintering (SPS) and chemical vapor deposition (CVD) have successfully fabricated pure W parts, manufacturing complex shapes and large sizes is challenging. Processing of pure W by additive manufacturing (AM) has received increased attention due to a significant potential to produce parts from materials that could not be processed by traditional methods^2^. One type of AM technique is Laser Powder Bed Fusion (PBF-LB), where higher cooling rates in comparison with other traditional and AM techniques are seen. Extensive cracking of tungsten deposited by PBF-LB has been reported^3–9^.

While the exact cracking mechanisms in pure W are not well understood, the generally observed cracking mode for pure W fabricated by PBF-LB is transverse to the laser scanning direction. The cracking mechanism is termed “delayed cracking”, caused by thermal stresses combined with the temperature falling below the DBT^10–12^. It has been suggested that the formation of brittle tungsten oxides at the grain boundaries contributes to intergranular cracking^4,7,13^.

**Fig. 1:**
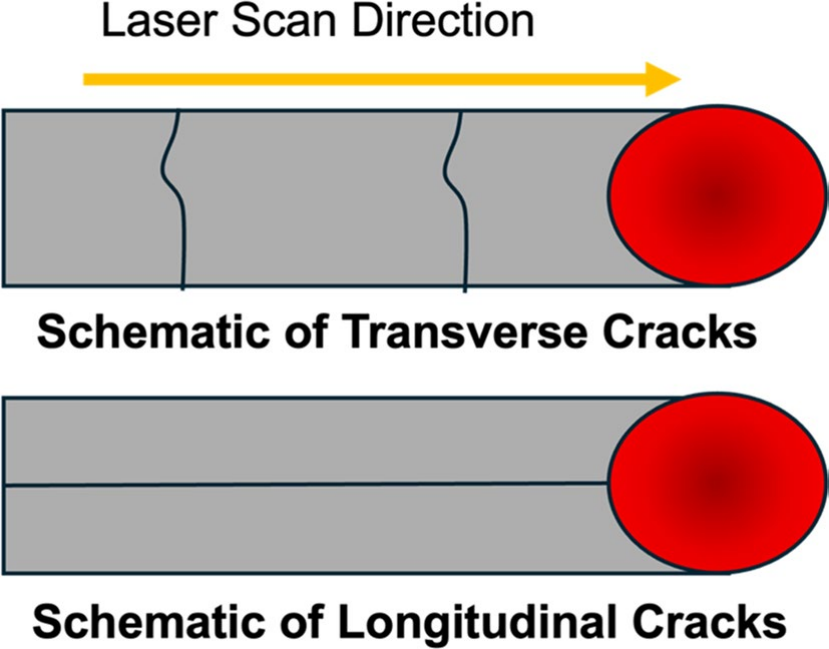
Schematic of transverse and longitudinal cracks seen in PBF-LB melting of Pure W.

*Longitudinal cracks* along the scan direction, have also been observed^5,6,11,12,14^. Figure 1 shows a schematic of transverse and longitudinal cracks seen in PBF-LB of pure W. Vrancken *et al.* observed longitudinal cracks at higher powers and attributed them to deeper melt pools and unfavorable grain orientations resulting in stress relief by cracking along the centerline of the melt pool^11^. Chen *et al.* performed single track experiments and finite element analysis to conclude that longitudinal cracking is related to the unfavorable grain orientations at high melt pool depths^5^. In this work, the possibility is examined that these may be solidification cracks, formed during the final stages of solidification.

Solidification cracking (also known as hot cracking) occurs towards the end (~the last 2-5 %) of solidification, when the material is close to the solidus temperature^15,16^. This type of cracking typically manifests as longitudinal cracks along the centerline of the weld track and intercellular cracking within the microstructure. The metallurgical factors that affect this type of cracking are:

1. Large freezing range
2. Presence of low melting eutectics
3. Surface tension of the grain boundary liquid.

**Fig. 2:**
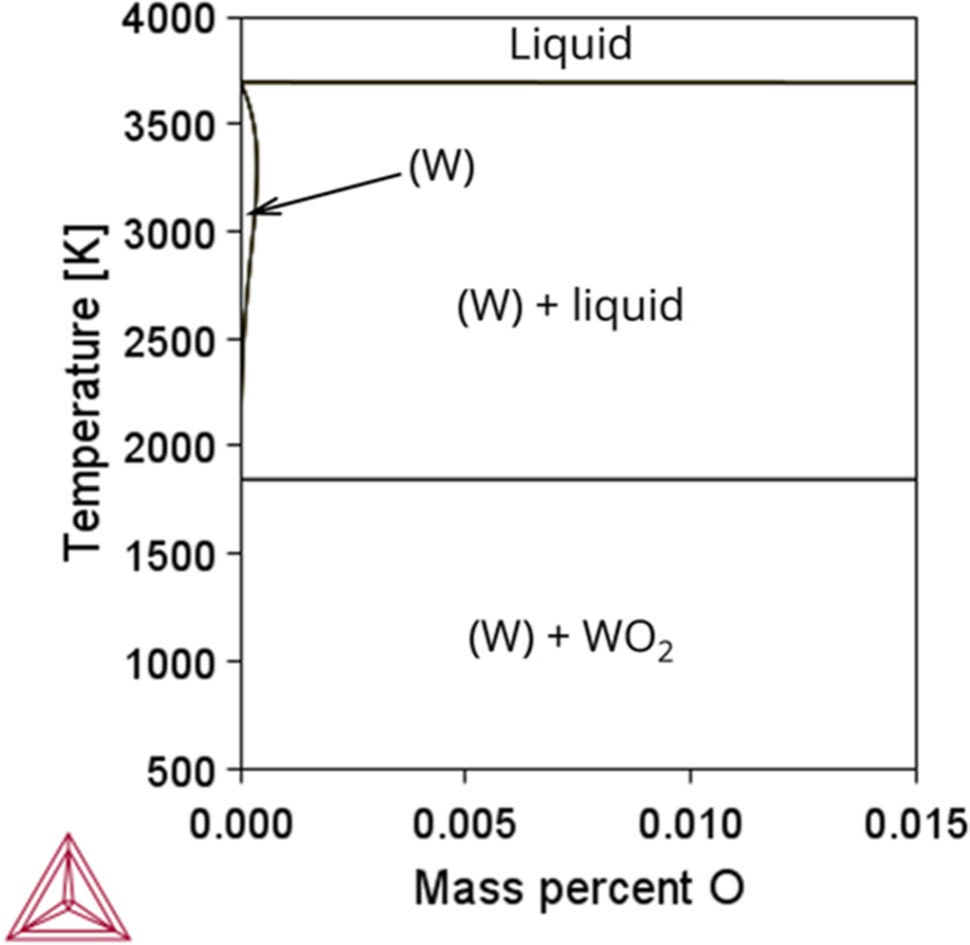
Calculated W–O phase diagram based on Thermo-Calc (version 2024b, TCOX13 database). Limitations and applicability of this diagram are discussed in the text.

While pure W melts congruently, the presence of impurities and interstitial elements in commercial purity W base plates can increase this freezing range, potentially contributing to solidification cracking. To illustrate the possible effect of even low concentrations of oxygen, Fig. 2 shows the W-O phase diagram computed using the TCOX13 database of Thermo-Calc (version 2024b)^17,18^. The predictions show very limited solubility of O in W, as well as a large difference between the liquidus (3430 °C) and solidus temperatures (1582 °C) for alloys with more than 15 ppmw of oxygen.

The actual solidus temperature must be higher, since unmelted W_18_O_49_ was reported at 1700 °C^19,20^ and this phase is not included in TCOX13. There is still uncertainty above the very high temperature region of this diagram, but all the available data indicate very low solubility of oxygen in solid tungsten at all temperatures, with strong partitioning of oxygen to the remaining liquid during solidification. The appearance of liquid oxide below the melting temperature of the pure metal would support the hot-cracking requirement of relatively low-melting liquid remaining after most of the alloy has solidified.

Another observation supporting the suggestion that oxygen can contribute to hot cracking was made by Braun *et al.*, who found oxides at the grain boundaries and fracture surfaces of molybdenum (Mo) and tungsten deposited by PBF-LB. The presence of residual liquid towards the end of solidification was ascribed to the low solubility of oxygen in Mo; evidence was shown of oxide precipitates forming by eutectic solidification in Mo and W. The authors proposed hot cracking as a potential cracking mechanism in Mo and suggested that similar behavior may be observed in W^6^. Field *et al.* proposed that longitudinal cracks were caused by embrittlement due to oxygen segregated at the grain boundaries in W^14^. Furthermore, Randhavan *et al.* proposed that solidification cracking occurred during single-track and pad experiments; however, the role of oxygen in the powder and the build atmosphere was not investigated^21^.

This work explores the metallurgical factors that cause longitudinal cracking in W and utilize single tracks in PBF-LB systems to explore the possibility of hot cracking. Additionally, in situ synchrotron X-ray radiography of W is performed to investigate meltpool behavior and cracking. The rationale for the experiments was to identify cracking phenomena for single tracks melted at different combinations of power and velocity, in two laser-melting systems with different oxygen activities in the build atmosphere. Synchrotron imaging—which is highly challenging for a high-density material like tungsten—was used to identify possible solidification cracking. For all three types of experiment, the solidified material was examined to identify the types of cracks, the grain structure, and possible evidence for a role of oxygen.

## Results

The results in the following sections are divided into the following sections: (1) Build Images, (2) Top-Down SEM Images, (3) Meltpool Cross-section Images, (4) Meltpool Geometries, and (5) in situ Synchrotron X-ray Radiography.

### Build images

**Fig. 3:**
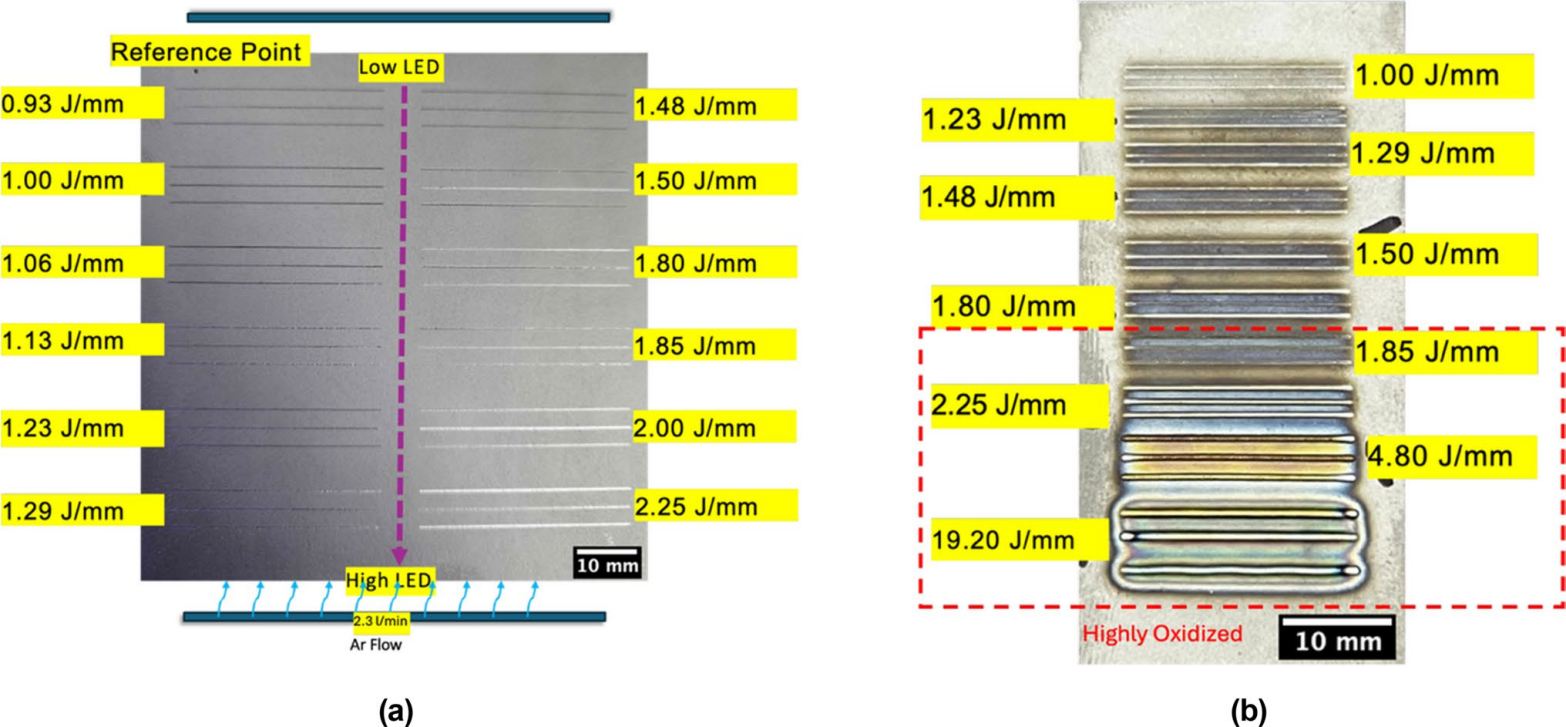
Build images of single tracks obtained from (**a**) Trumpf PBF-LB machine, operating under Ar atmosphere (840–850 ppmv oxygen) and (**b**) Custom PBF-LB machine, operating under vacuum (equivalent to less than 250 ppmv oxygen).

Figure 3 shows the build images of the single tracks after printing as follows: Fig. 3a shows the build plate after single-track melting in the Trumpf PBF-LB machine. No visible oxidization can be seen on the build plate surface although there was approximately 850 ppmv of oxygen in the build chamber during single track melting process. In contrast, the build plate after single-track melting in the Custom PBF-LB machine in 0.9 torr low vacuum showed surface oxidation on the melt tracks (Fig. 3b), more so in the lower half of the build plate, which has the wider tracks melted at higher LED towards the end of the melting tests.

### Top-Down SEM images

**Fig. 4:**
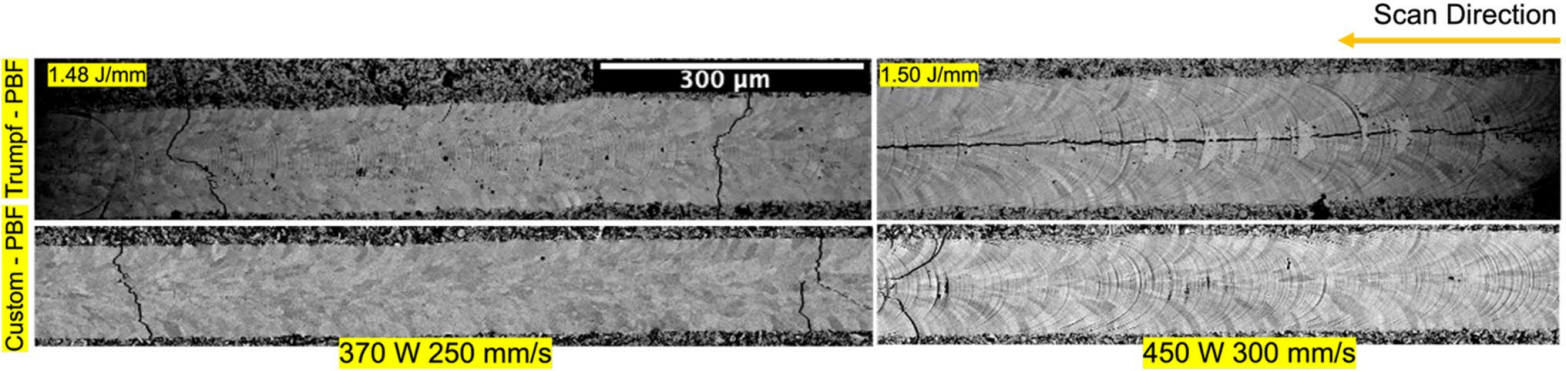
Comparison of top-down views of tracks of 370W and 250 mm/s, and 450 W and 300 mm/s in the Trumpf PBF-LB and custom PBF-LB machines.

Figure 4 compares top-down single-track images for tracks produced in the two PBF-LB systems at similar linear energy density. Note that while an equiaxed grain structure is formed at 370 W, columnar grains structure are seen in 450 W power tracks.

**Fig. 5:**
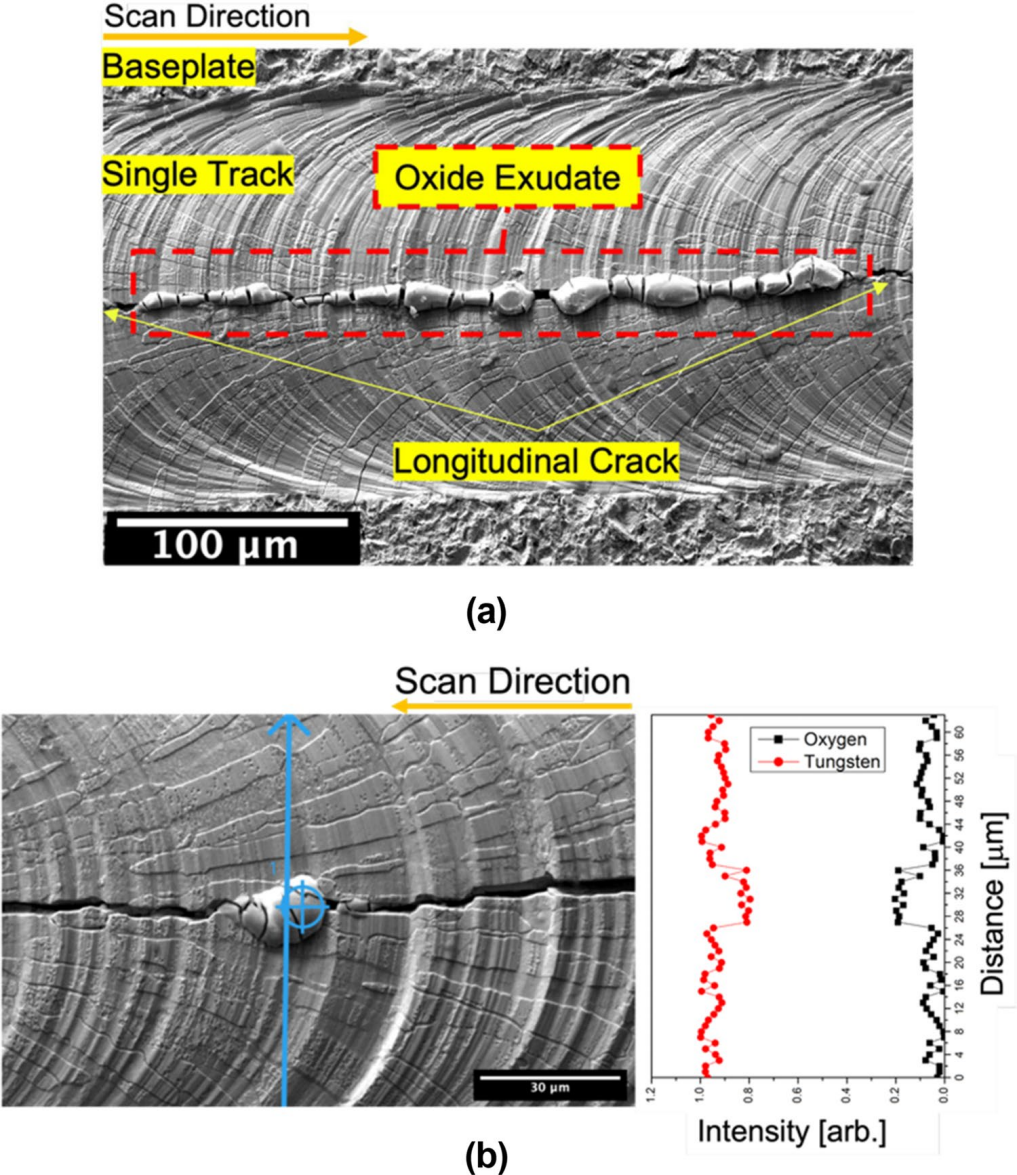
Representative top view of a longitudinal crack in a track molten in the Trumpf PBF-LB machine (850 ppmv oxygen in the build atmosphere) showing: (**a**) Secondary electron micrograph at 450 W and 250 mm/s and (**b**) Analyzed (EDS) weight fraction of oxygen along a line crossing the exudate obtained at 450 W and 300 mm/s.

Longitudinal cracks were seen for tracks produced at 450 W in the Trumpf - PBF system; see Fig. 5a for a representative image of such longitudinal cracking (track produced at 450 W and 250 mm/s). Features can be seen exuding from the cracks. These exudates were not observed in any 370 W single tracks (nor was longitudinal cracking observed for the 370 W tracks).

Energy-dispersive X-ray spectroscopy (EDS) of the exudates shows that these are W oxides (Fig. 5b; track melted at 300 mm/s), with an increase in oxygen concentration along the exuded features.

**Fig. 6:**
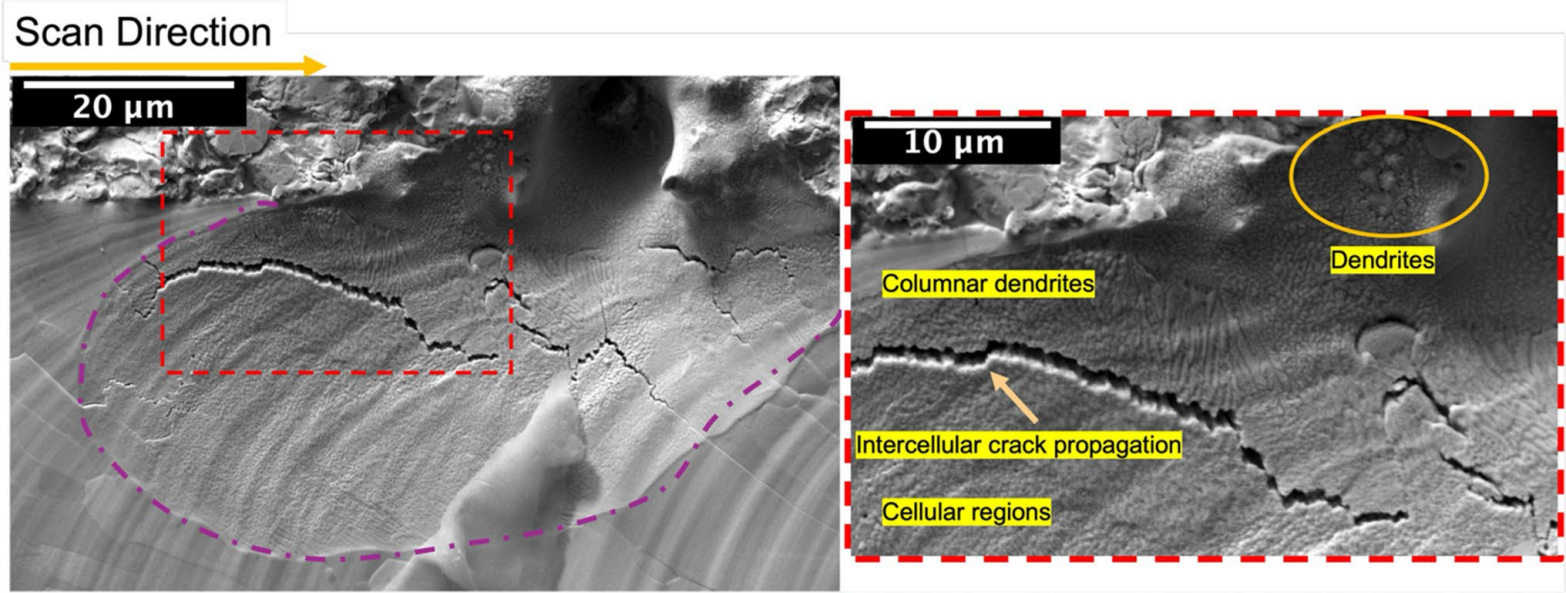
Representative top-down image near the edge of a single track showing solidification microstructure features and associated cracks, for the custom PBF-LB machine. The image shown is for a laser power of 370 W and a 250 mm/s scan speed.

The custom PBF-LB single tracks did not show any oxide exudates from the cracks. However, as seen in Fig. 6, the edges of the melt track showed several shallow concavities - one of which is marked with broken purple lines in the image (track produced at a laser power of 370 W and scan speed of 250 mm/s, imaged in SE mode). Additionally, these concavities show cellular regions and dendrites. These features are present in all the single tracks printed using the custom PBF-LB machine. Cells and dendrites cannot form in pure metals under constrained-growth conditions, and are caused by the partitioning of elements between the solid and liquid; the presence of oxygen (that partitions strongly to the remaining liquid) is a likely cause of these solidification structures. The observation of both cellular and dendritic structures likely reflects local differences in solidification rate in different regions of the melt pool.

**Fig. 7:**
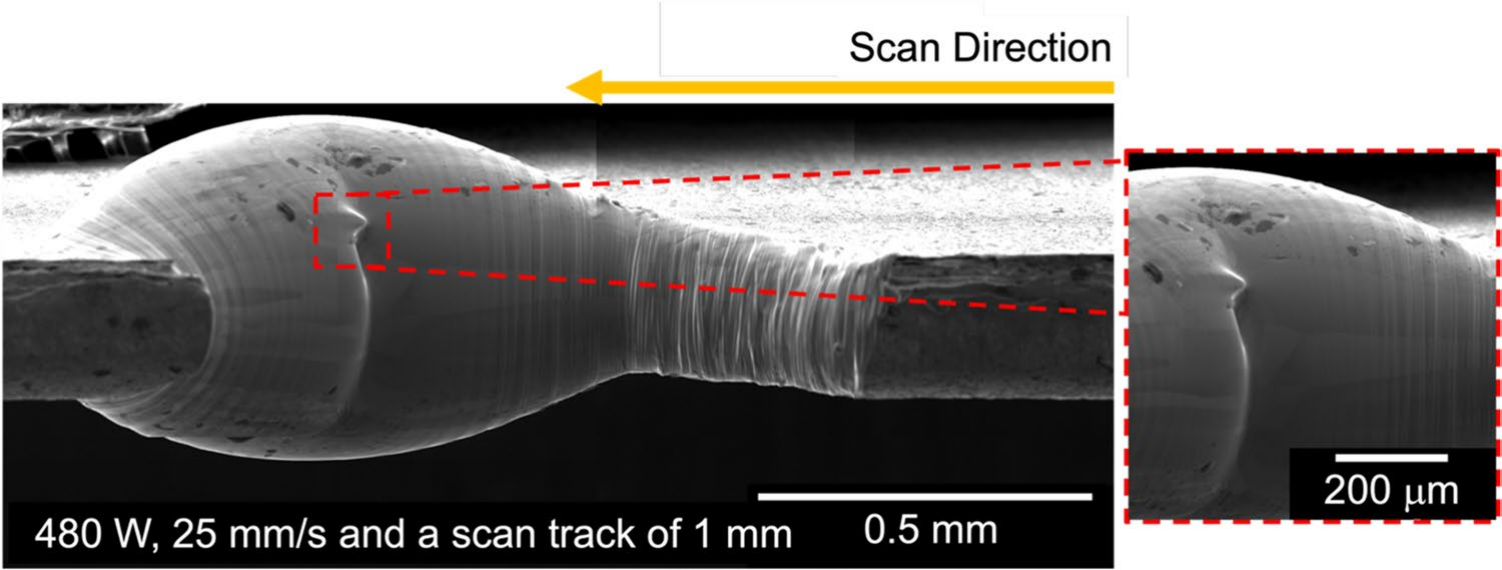
Top-down view (secondary electron micrograph) of the 1 mm long scan track and the higher magnification around the crack region at 480 W and 25 mm/s in the Operando setup.

Figure 7 shows a secondary electron micrograph of the scan track melted at 480 W and 25 mm/s in the in situ synchrotron X-ray radiography setup. While no oxide exudates or shallow concavities are observed, the swelling of the material can be observed. Due to the high density of tungsten, a sample thickness of 0.15 mm was used. Some slumping of the melt pool occurred owing to the 0.1 mm beam diameter and keyhole conditions, resulting in a large thickness (“swelling”) in the melt-pool region. Cracking was observed at the end of the laser track, evident in the magnified image.

### Cross-section SEM images

**Fig. 8:**
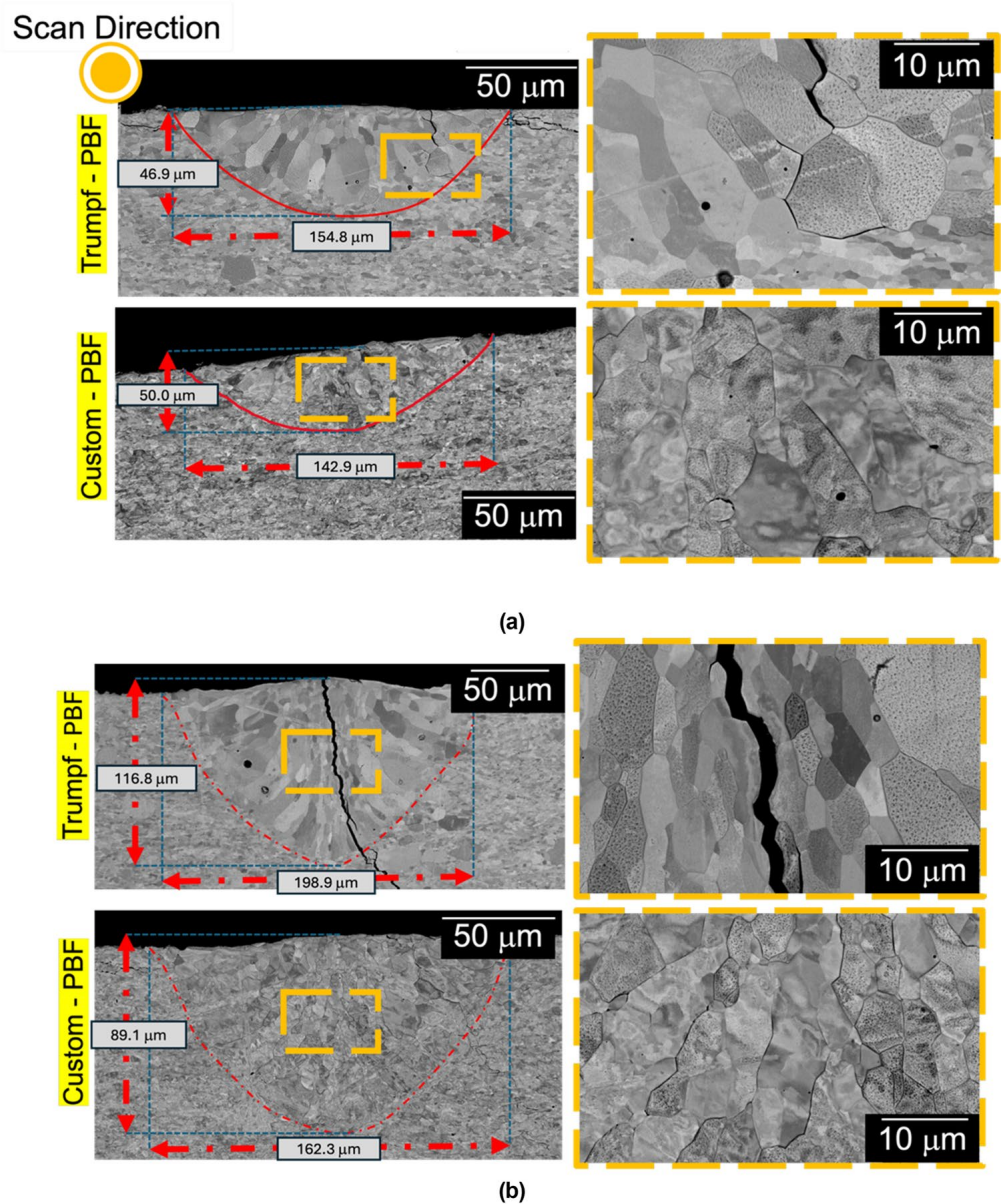
Comparison of melt pool cross-sections for tracks prepared in the Trumpf and custom PBF-LB machines for (**a**) 370 W and 250 mm/s and (**b**) 450 W and 300 mm/s. The images on the right are higher-magnification images from the selected region. The etched samples are imaged in BSE mode. Intergranular cracking is evident in the 450 W track melted in the Trumpf-PBF system. Note that the cracking shown in the 370 W track melted in the Trumpf-PBF system is likely the Y–Z cross-section of a transverse crack in the X–Y scan direction.

Scanning Electron Microscopy (SEM) images of single-track cross-sections in Fig. 8 (Trumpf PBF-LB and custom PBF-LB systems) do not show longitudinal cracks for tracks produced at 370 W and 250 mm/s (Fig. 8a). Tracks melted at 450 W and 300 mm/s show longitudinal cracks for the Trumpf PBF-LB but not the custom PBF-LB (Fig. 8b). Compared with the top-down images, grains appear more equiaxed in the cross-sections. Differences in cracking behavior between the melting systems when using the same power and speed support the suggestion of a role of oxygen in cracking.

**Fig. 9:**
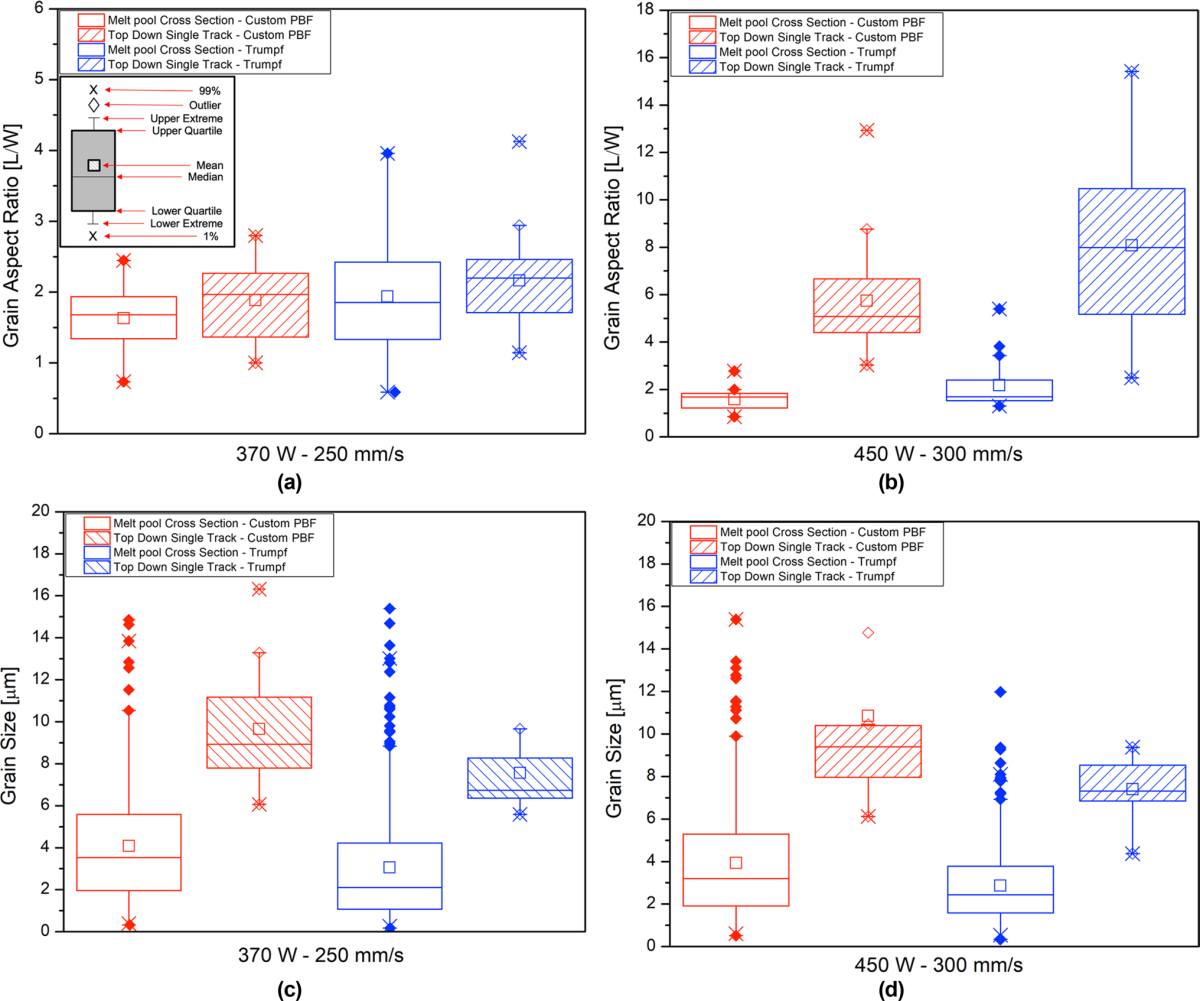
Comparison of grain aspect ratio and grain size for tracks produced at 370 W and 250 mm/s and 450 W and 300 mm/s. (**a**), (**c**) show the grain aspect ratio and grain size for tracks produced at 370 W and 250 mm/s. (**b**), (**d**) show the grain aspect ratio and grain size for tracks produced at 450 W and 300 mm/s.

The similarity of the melting conditions between the two melting systems is also shown by quantification of the grain size and aspect ratio for single tracks produced in the two systems (Fig. 9). For tracks produced at 370 W and 250 mm/s (Fig. 9a,c) the aspect ratios are similar, both for the two orientations and between the two systems; the only difference is that the mean grain size is larger in the top-down single track images (for both systems). Similar observations hold for tracks produced at 450 W and 300 mm/s (Fig. 9b,d), with larger grains in the top-down single track images, but also a larger grain aspect ratio in the top-down view for both systems. The higher aspect ratio agrees with the Fig. 4 which shows the presence of columnar grain growth towards the melt pool scan direction.

**Fig. 10:**
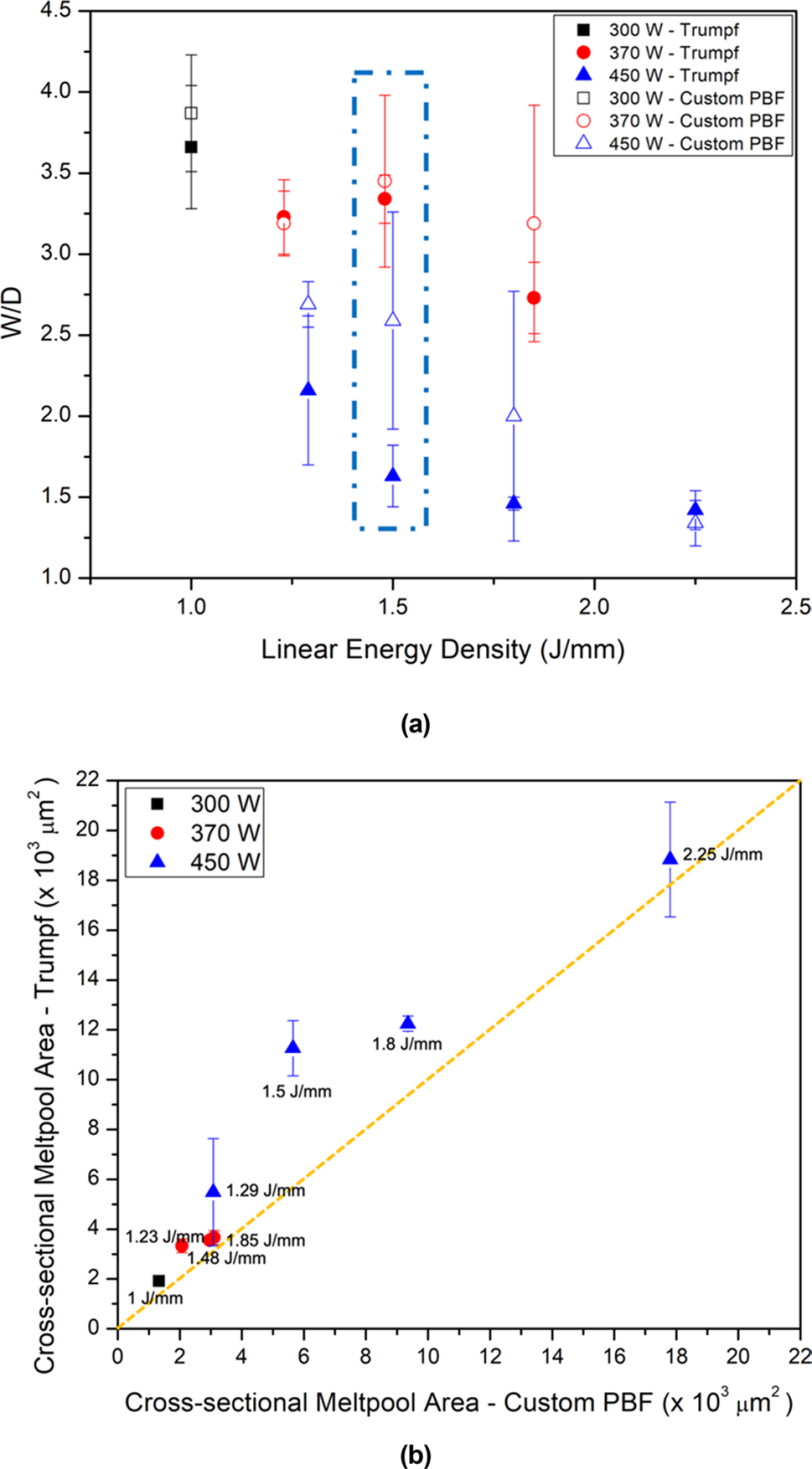
Melt-pool aspect ratio and cross-sectional area for Trumpf and custom PBF-LB machine is shown where (**a**) shows the change in melt-pool aspect ratio with linear energy density for tracks melted in the Trumpf and custom PBF-LB machines. The blue dotted box represents the tracks shown in this paper. The error bars indicate the standard deviation in the melt-pool aspect ratio as measured from 3 different single tracks and (**b**) comparison of melt-pool cross-sectional area between the Trumpf and custom PBF-LB machines.

Comparison of melt-pool aspect ratios and sizes shows slightly lower W/D ratios (ratio of melt-pool width to depth) for the Trumpf tracks (Fig. 10a), with slightly larger melt-pool cross-sections (Fig. 10b). The larger melt pools were likely caused by shorter delay times between tracks in the Trumpf machine, causing a higher preheat.

### In situ synchrotron X-ray radiography

**Fig. 11:**
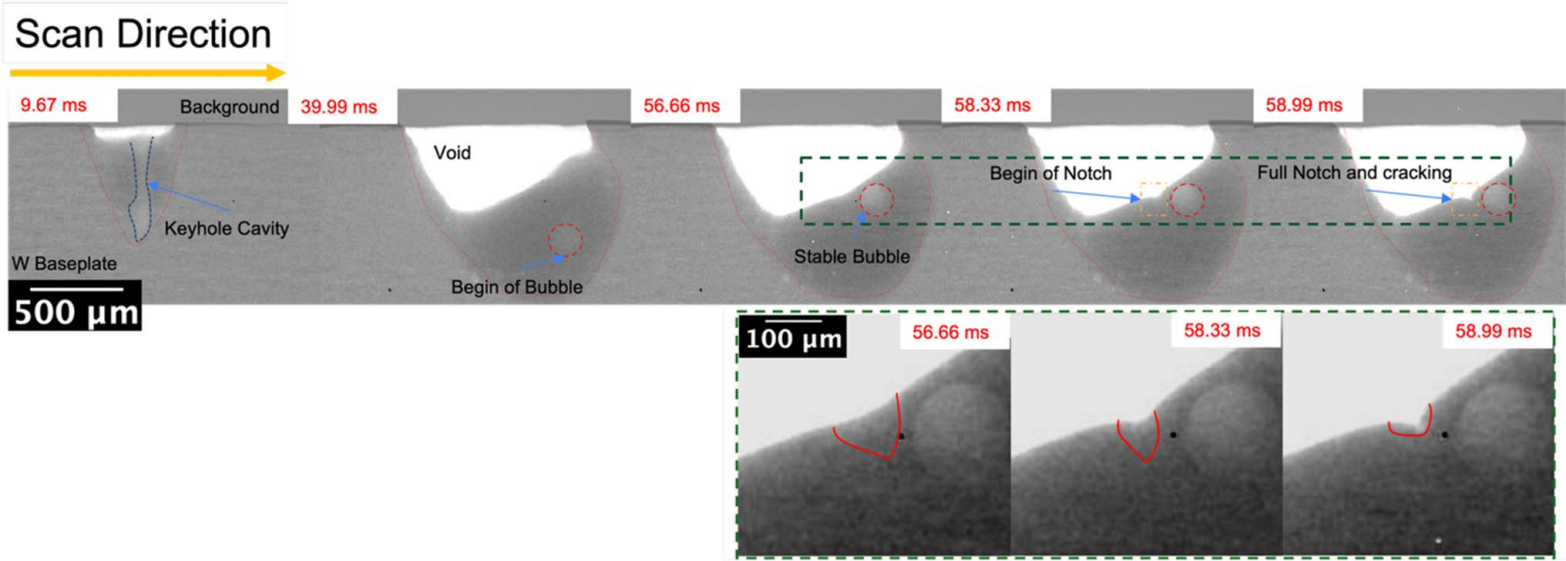
Selected images from a times series of high-energy synchrotron X-ray radiography for the laser conditions of 480 W and 25 mm/s. The images show the formation of a keyhole, bubble after the collapse of the keyhole , bubble travel to the top of the meltpool, notch initiation, and cracking. The images in the green box are higher magnification from the regions highlighted.

Figure 11 shows a montage and processed images from selected frames. The images in Fig. 11 show a keyhole mode melt pool with a void - as seen by the white space in the still images. The void is likely a result of the slumping (overflowing) melt pool, also evident in Fig. 7. After the laser was turned off, a vapor bubble could be observed traveling to the top of the melt pool. Oscillatory movement of the bubble continued for time. Due to the dense nature of W, a solid-liquid interface cannot be distinguished; instead, the onset of solidification was identified by the cessation of bubble movement. A crack was subsequently observed, starting from the base of the depression left by the void. The full video of the laser scan track can be found in the supplementary material.

**Fig. 12:**
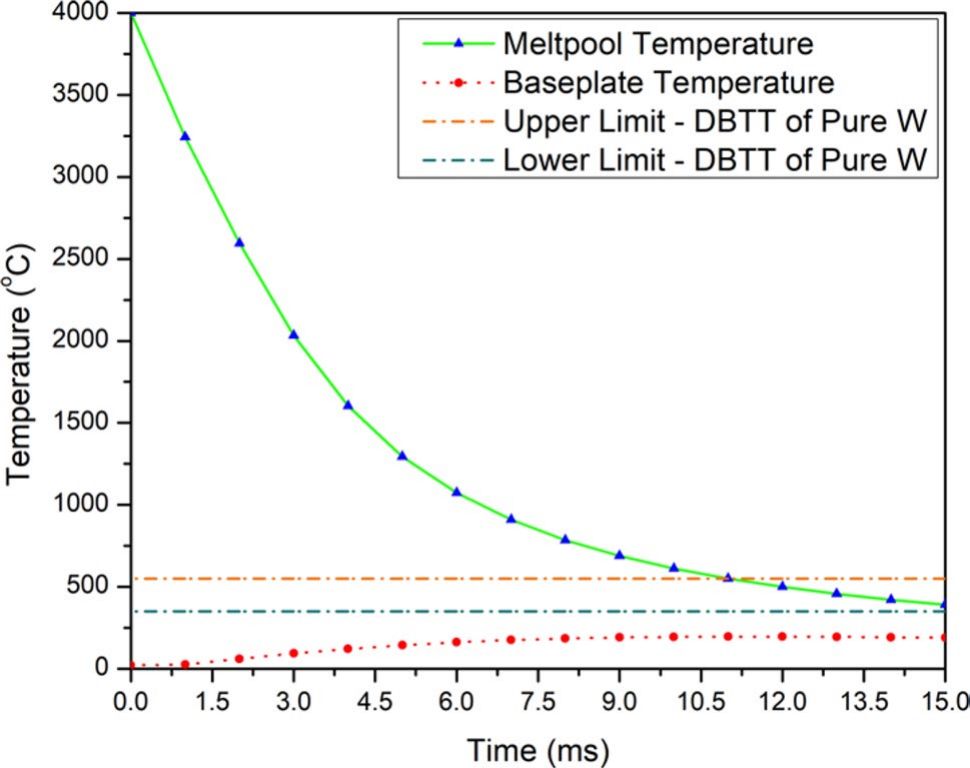
Calculated cooling of the melt pool under the synchrotron imaging conditions. Note that the Upper and Lower DBTT limits of Pure W are obtained from literature^2,11,22,23^.

Table 1 summarizes various key events from the radiography montage, with approximate time stamps. Based on the radiography images, the time from solidification (cessation of bubble movement) to the beginning of cracking was ≈7 ms. In comparison, the estimated time for the melt pool to cool from 4000 °C to below the DBTT was around 10 ms (Fig. 12), similar to the time interval from solidification to cracking.

**Table 1:** A summary of various key events from the radiography montage and the approximate time stamp (ms) for a 1 mm single track produced at 480 W and 25 mm/s.

Event	Approximate time stamp (ms)
Last keyhole cavity appearance—Laser stop	40.00
Stable Bubble (considered the onset of solidification)	52.00
Beginning of crack	58.67
Final Crack	59.67
**Interval **	**Time difference (ms)**
Solidification to crack formation (Stable bubble until beginning of crack)	6.67
Crack Propagation (beginning of crack to final crack)	1
Time from laser stop to final crack	19.67

**Fig. 13:**
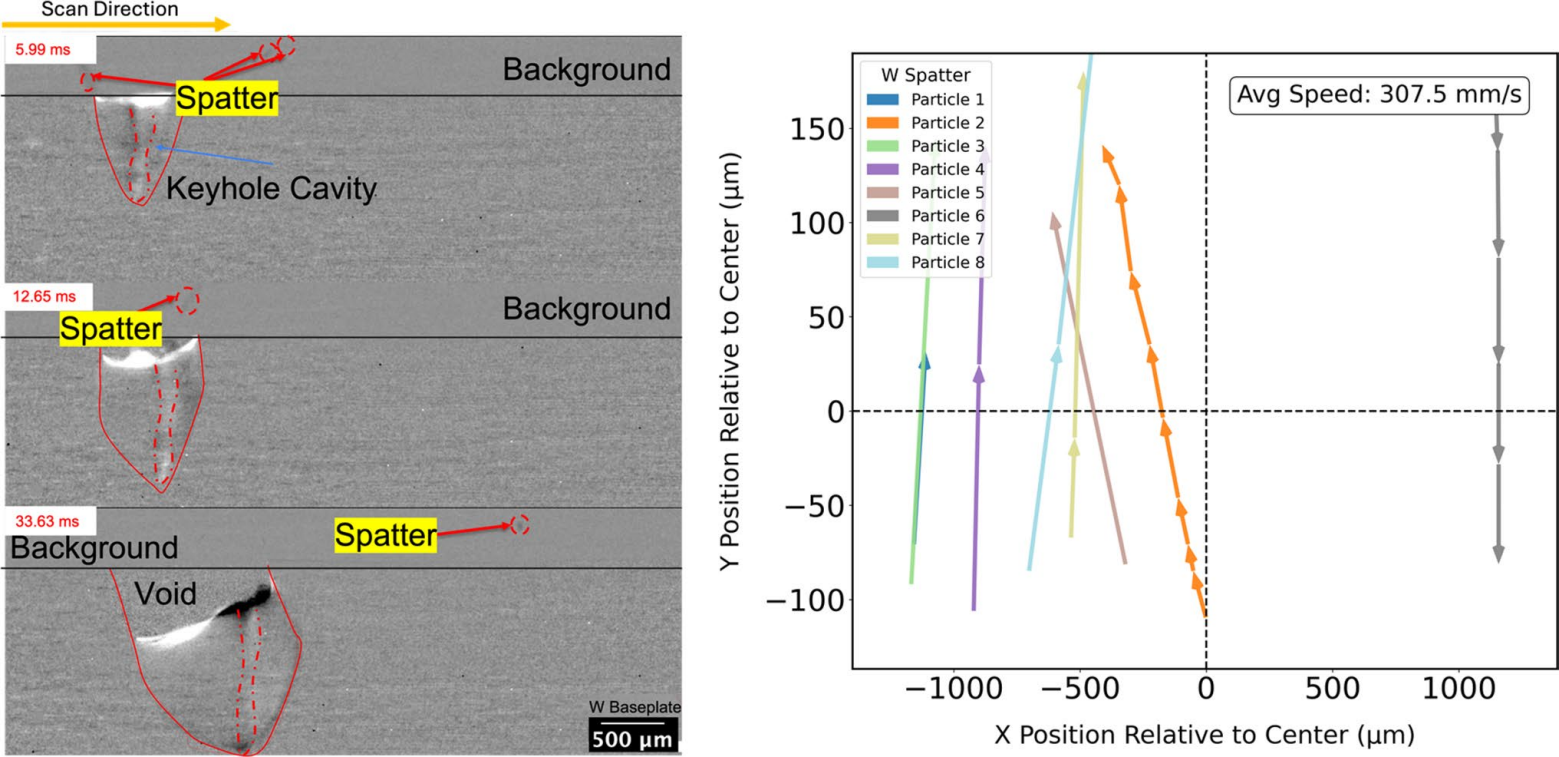
Selected processed images from a times series of high-energy synchrotron X-ray radiography for the laser conditions of 480 W and 25 mm/s. The processed images show the spatter events and spatter landing back on the baseplate. The quiver plot shows the spatter directions from the meltpool for different particles at different stages during the melting along with a spatter particle falling back to the baseplate. The average speed of the spatter has been tracked and is labeled.

Hot spatter is a mechanism for oxygen pick-up by the meltpool. The synchrotron imaging allowed spatter to be tracked: select frames (Fig. 13) show both spatter produced from the melt pool, and spatter landing back on the baseplate. In the same figure, the quiver plot of tracked spatter particle trajectories shows the nature of spatter travel in pure W PBF-LB melting.

### Estimated direct oxygen pickup by liquid meltpool

**Fig. 14:**
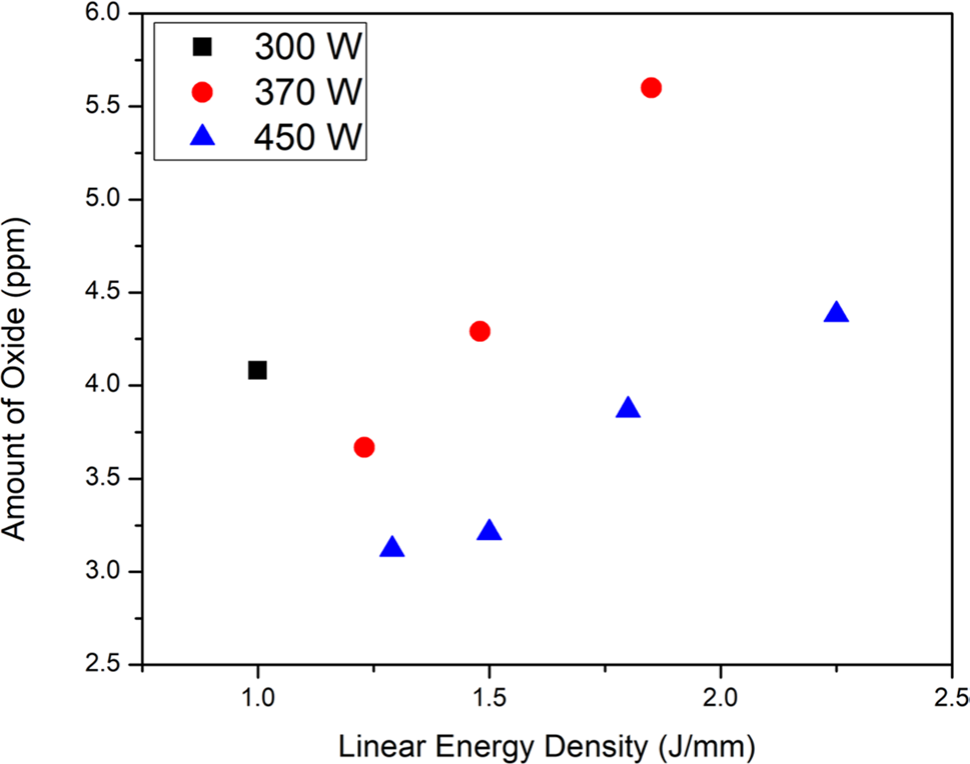
Calculated oxide pickup by the melt pool directly from the gas atmosphere, for tracks prepared with different P–V combinations in the Trumpf system.

Figure 14 shows the estimated oxygen pickup by the liquid melt pool directly from the gas atmosphere for Trumpf PBF-LB (higher oxygen activity) conditions; the maximum estimated pick-up is approximated 6 ppm, which is small compared with the starting oxygen concentration (in the base plate) of 30 ppm. This supports the suggestion that spatter—rather than direct oxidation—is a significant oxygen transfer mechanism.

## Discussion

The results from three different melting conditions show that cracking in pure W following laser melting is influenced by several factors. The results support the suggestion that solidification cracking can occur for commercial-purity tungsten in some cases. The tungsten-oxygen phase equilibria at high temperatures are not fully understood, but it is possible a liquid oxide phase is present. Thus, sufficient melt pool oxygen uptake may cause solidification cracking. This is supported by the appearance of longitudinal cracks with exuded oxide in Trumpf PBF tracks—with high oxygen activity and Ar flow perpendicular to the scan track. In the Custom system where tracks were deposited under vacuum (lower oxygen activity), no longitudinal cracks were observed, only intercellular cracking at the edges of scan tracks.

Longitudinal cracking was not observed in tracks melted in the custom PBF-LB system. Given that oxygen is a likely source of the liquid that can cause solidification cracking, the absence of cracking for the custom PBF-LB melts is consistent with the expected lower oxygen activity in the custom PBF-LB build chamber: as discussed in the methods section, even if the entire chamber pressure of 0.9 torr were due to entrained air, the oxygen activity would be equivalent to 1 atm gas with an oxygen concentration of 200 ppm. This is less than a quarter of the oxygen activity that prevailed in the Trumpf PBF-LB chamber, leading to a lower amount of oxygen available to react with meltpool spatter.

The observed oxidation of the custom PBF-LB base plate—unlike the plate from the Trumpf machine—appears to contradict the expected less-oxidizing conditions in the custom PBF-LB system. However, the surface oxidation likely occurred after printing, when the chamber was vented to atmosphere, rather than during melting of the tracks.

Based on literature calculations, the main mechanism of oxygen transfer to the melt pool is by the oxidation of hot spatter that falls onto the base plate ahead of the laser, or directly onto the track^24,25^. This phenomenon can also be observed in Fig. 13. Increased transfer of oxygen to the melt pool is also a possible reason for the observation of cracking for the Trumpf-system tracks produced at 450 W, but not at 370 W: More spatter would have been produced at 450 W^26^, increasing oxygen transfer to the melt pool. In addition, the tracks prepared at 450 W were larger and deeper (with a smaller W/D ratio), likely increasing the residual stress in the solidified material. Care should be taken when comparing the grain sizes for these conditions: while the grains might appear equiaxed on a cross-section as seen in Fig. 8b, the plan view of the track shows that the grains are in fact columnar, growing opposite to the direction of heat extraction; similar observations were made by Wei et al. based on Monte-Carlo simulations of 3D grain growth in a weld pool, about the columnar vs equiaxed discrepancies^27^.The grain sizes in plan view were larger for the 450 W tracks (Fig. 9d) than for the 370 W tracks (Fig. 9c), which could have further contributed to cracking of the 450 W tracks. Although alternatively, cracking along the grain boundaries can be caused by brittle oxides that formed in the last remaining liquid, the presence of oxide exudate in the Trumpf system and intercellular crack propagation in the custom PBF-LB system all suggest solidification cracking as a more likely mechanism.

Ductility-dip cracking is another potential failure mechanism, generally observed between 0.5 times and 0.9 times the absolute melting point, and has been observed in pure tungsten as a decrease in ductility^28^. However, the crack morphologies observed here do not show the grain-boundary sliding that is generally associated with ductility-dip cracking; instead, these cracks are consistent with brittle cracking (transverse cracks) and solidification cracks (longitudinal cracks)^29^.

Cracking of the sample melted during synchrotron imaging cannot be attributed to hot cracking: The crack formed approximately 7 ms after solidification, by which time the temperature is estimated to be below 1000 °C, well below the solidus temperature for oxygen-containing tungsten. While there might be some error in the time estimates due to the radiography frame rate, it is apparent that the cracking of the synchrotron sample was by brittle failure below the DBT temperature. This also agrees well with the scan track (Fig. 7) where a transverse crack could be observed at the end of the scan track.

In conclusion, solidification cracking in W fabricated via PBF-LB powderless single tracks is discussed. Evidence is presented for solidification cracking of commercial-purity W under PBF-LB conditions:

- For tracks melted at a higher oxygen activity (with around 800 ppmv oxygen in the 760 torr build atmosphere), longitudinal cracks were observed, with oxides exuding out of the cracks—likely the liquid remaining during the last stages of solidification. Longitudinal cracks were only observed for the highest power tested (450 W), indicating possible effects on cracking of oxygen transfer by spatter, and of melt-pool shape and grain size.
- For tracks melted under vacuum (0.9 torr), no longitudinal cracks were observed. However, localized cracking was observed near the edges of the melt pools, with intercellular crack propagation.

The results support solidification cracking, (manifesting as longitudinal cracks) as an additional failure mechanism, in addition to brittle cracking below the DBTT (that manifest as transverse cracks). This work shows the application of high-energy synchrotron X-ray radiography on a dense material like W. Useful future work would involve utilizing this technique and other high-speed imaging techniques to study spatter and its effects in laser melting of W. Furthermore, high-temperature experiments are required to understand the W-O interactions that can aid in better phase diagram generation.

## Materials and methods

### Materials

Two commercially pure W plates were used to study longitudinal cracking during single-track remelting in a commercial PBF-LB and a low-vacuum custom printer are discussed. The plates—prepared by pressing and sintering according to ASTM B760^30^—were obtained from Midwest Tungsten. The plates contained 30 ppm of oxygen by mass. For the Trumpf PBF-LB machine, a 88.9 mm (L) × 88.9 mm (W) × 6.35 mm (H) baseplate was used; the baseplate for the custom PBF-LB printer measured 88.9 mm (L) × 31.75 mm (W) × 6.35 mm (H). The baseplate size was 10 mm (L) × 0.15 mm (W) × 10 mm (H) for the in situ high speed synchrotron X-ray radiography.

### Methods

Given the nature of PBF-LB, oxygen intake can originate from both the powder feedstock and the build atmosphere. To understand the role of oxygen on cracking mechanism, single track experiments without the use of powder were conducted in the following three different PBF-LB setups utilizing the same laser wavelength and operated under identical laser power and scan velocity parameters, allowing for a controlled comparison aimed at evaluating the influence of oxygen on cracking:

1. Trumpf TruPrint 3000 PBF-LB machine (Ditzingen, Germany) is equipped with a fiber laser of 1070 nm wavelength, maximum power of 500 W and variable beam diameter. For this study, a 100 µm laser beam diameter was used. Ar flowed perpendicularly to the laser scan tracks at 2.3 L/min. While no inter-scan track delay was set, it was the time taken for the laser optics to adjust for the change in scan direction between each scan track. The oxygen levels in the argon atmospheres during the experiments were 840–850 ppmv during printing, as monitored by the oxygen sensors in the PBF-LB machine.
2. A custom-built PBF-LB machine developed by Lawrence Livermore National Laboratory was used for melting experi- ments under low-vacuum conditions, with an Yb-doped fiber laser of 1070 nm wavelength and maximum power of 1 kW. This build chamber was prepared by pumping to 0.9 torr for 45 min prior to printing and continuously maintaining a build chamber pressure of 0.9 torr throughout the experiments. No Ar flow was utilized. The oxygen activity was not measured, but if the pressure of 0.9 torr were entirely from air ingress, the oxygen activity would have been equivalent to the partial pressure of 760 torr gas containing 250 ppmv oxygen. No inter-scan track delay was set; each single track position was manually advanced, resulting in an increased time between each single track.
3. In situ synchrotron X-ray radiography experiments were performed at ID 19 at the European Synchrotron Radiation Facility. A Photron SA-Z high-speed camera with a frame rate of 3000 images/s lense-coupled to a LuAG:Ce single- crystal scintillator with a nominal pixel size of 4 µm was used as an indirect detector for the high-speed imaging. The incident X-ray beam struck the side of the plate while a laser beam (maximum power 500 W, operating with a continuous wave fiber laser with a wavelength of 1070 nm and a beam diameter 100 µm) scanned across the field of view and melted the top of the plate. The laser scan speed was controlled with an SP-ICE-3 board and WeldMARK software (Raylase GmbH, Germany). The chamber was continuously flushed with Ar, with an oxygen level below 1000 ppmv. This miniaturized PBF-LB machine was custom built at the Paul Scherrer Institute, further details of the which can be found in^31,32^. In order to reach sufficient transmission with high-photon flux density for fast image acquisition, ID19 operated in white-beam mode where the light of the beamline’s wiggler (gap 26.5) was filtered by aluminium and copper attenuators to suppress softer parts of the emitted spectrum. Furthermore, compound-refractive lenses (CRLs) were placed in the X-ray optical beam path to further collimate the radiation in the detector field of view. The resulting mean X-ray photon energy of the impinging radiation was approximately 80 keV with a beam size of 4 × 4 mm^2^

Various combinations of power and scan speed were studied, resulting in a range of linear energy densities (LED) as defined by Eq. 1, where P is the laser power in Watts and V is the scan speed in mm/s.

1 $$LED = \frac{P}{V}$$

**Fig. 15:**
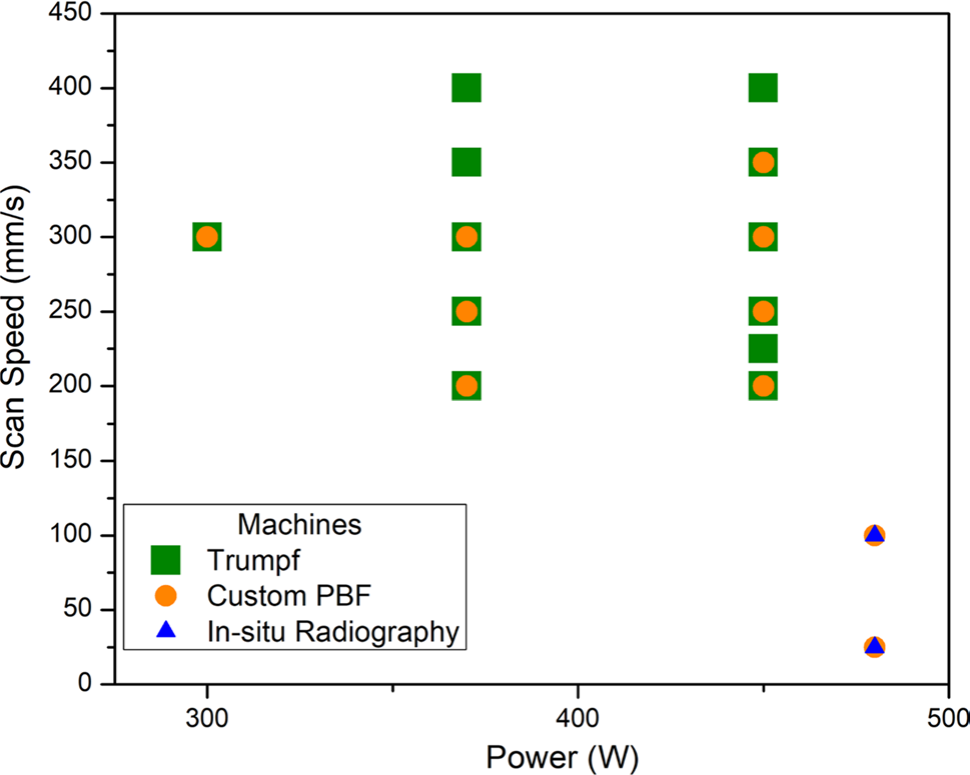
Process-Parameter space explored using the three PBF-LB techniques.

Table 2 and Fig. 15 show the process parameters used in the different PBF-LB machines. The scan tracks were 35 mm long and repeated 3 times per P-V combination for the Trumpf PBF-LB machine. For the custom PBF-LB machine, the tracks were 20 mm long and also repeated 3 times per P-V combination. In both the cases, the single track was rotated by 180° with no programmed cooling time between the passes. The scan tracks were 1 mm long in the in situ synchrotron X-ray radiography runs. The single track experiments were performed with no intentional preheat.

**Table 2:** Power and Scan Speed parameters used in the melting of nominally pure W using PBF-LB with a laser spot diameter of 100 µm.

Laser Power (W)	Scan Speed ( mm/s)	Linear Energy Density—LED (J/mm)	Trumpf—PBF-LB	Custom—PBF-LB	Synchrotron Radiography
370	400	0.93	x		
300	300	1.00	x	x	
370	350	1.06	x		
450	400	1.13	x		
370	300	1.23	x	x	
450	350	1.29	x	x	
370	250	1.48	x	x	
450	300	1.50	x	x	
450	250	1.80	x	x	
370	200	1.85	x	x	
450	225	2.00	x		
450	200	2.25	x	x	
480	100	4.80		x	x
480	25	19.20		x	x

### Estimated solidification time

**Fig. 16:**
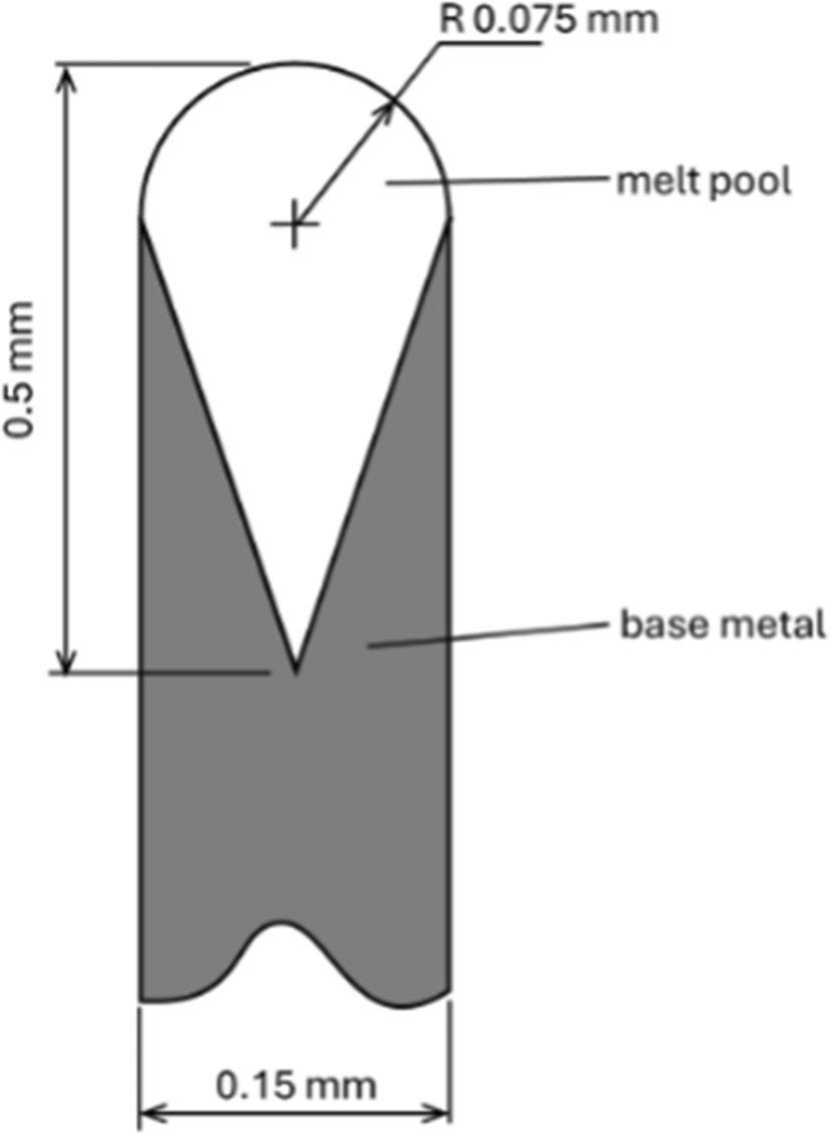
Modeled meltpool cross-sectional shape used for QuikCAST finite-difference simulation.

**Fig. 17:**
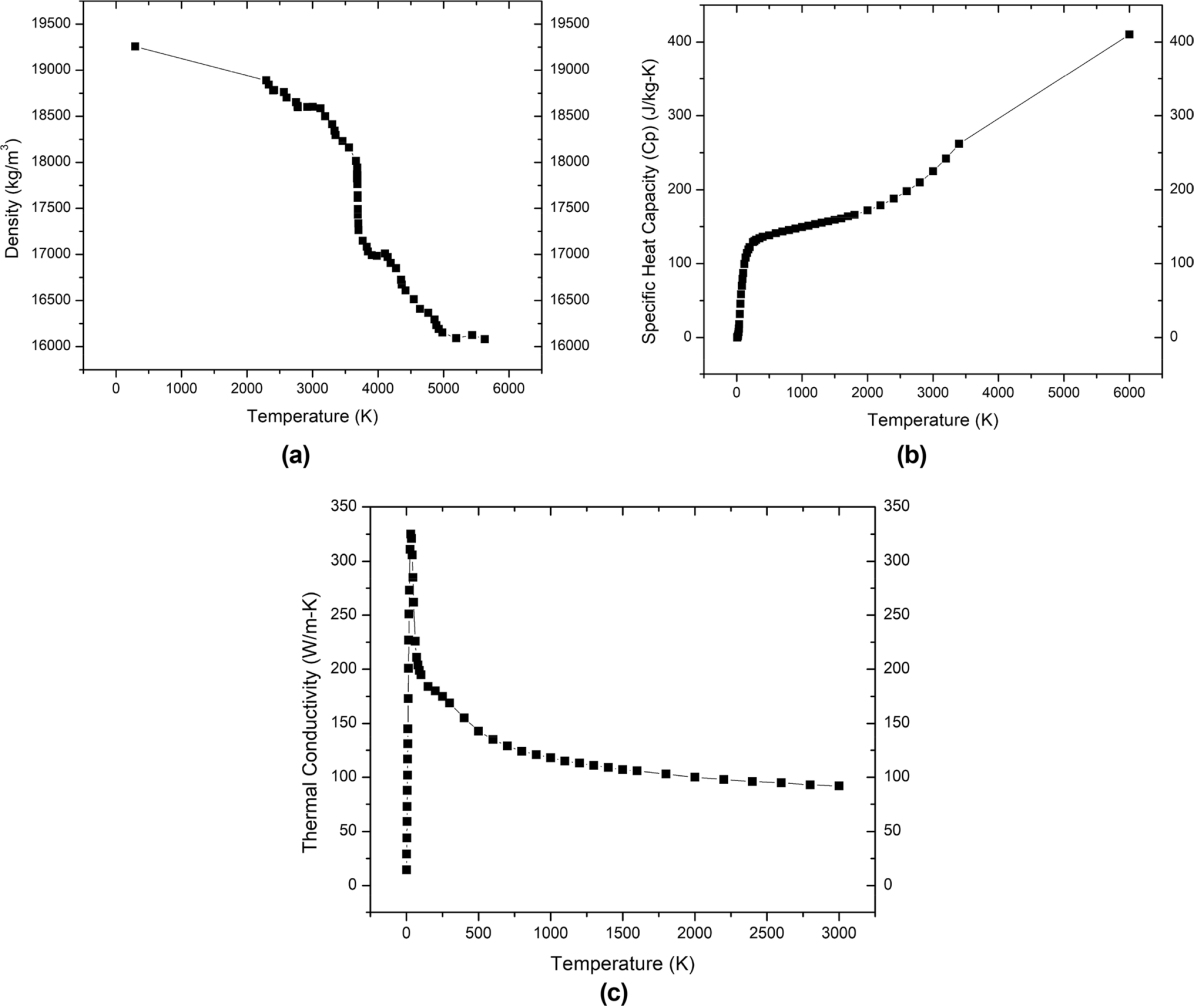
Temperature-dependent thermophysical properties of pure W, drawn using the literature data: (**a**) Density^35^; (**b**) Heat Capacity^34^; and (**c**) Thermal Conductivity^34^.

Cracks were observed following solidification in the synchrotron melting experiments. The temperature at which cracking occurred was estimated by simulating melt pool cooling with a QuikCAST finite-difference simulation; this is similar to the approach used in^33^. The modeled melt pool shape (dimensions in Fig. 16) was based on the measured melt pool. The melt pool was taken to be 1 mm long, embedded symmetrically in a base plate that was a total of 5 mm long and 2.8 mm tall (this height reflects the position of the plate in the sample stage during melting).Materials properties for pure tungsten were taken from^34,35^ and are summarized in Fig. 17.

The boundary conditions for the calculation were as follows:

1. At time zero, the melt pool volume was filled with liquid tungsten at 4000 °C, while the base plate was at 25 °C.
2. The bottom of the base plate remained at 25 °C while the top and sides of the base plate and the top of the melt pool exchanged heat with the 25 °C surroundings, through a combination of radiation (*ε* = 0.8) and convection (h = 10 W/m^2^K).

### Estimated oxygen pickup by the meltpool

Due to the limited available material volume inherent to single bead experiments and the brittle nature of oxides, it is nearly impossible to obtain sufficient sample mass that is distinct from the baseplate for direct oxygen content analysis. To address this limitation, we estimated possible oxygen pickup by the melt pool directly from the atmosphere in the build chamber, the mass transfer equations by Ohtuski et al. for IN 718 have been adapted to W^24,36^. The premise is that oxygen is transferred to the melt pool by argon flowing across the molten metal. The rate of oxygen transfer depends on the mass transfer coefficient for gas-metal reaction, and the oxygen concentration in the gas. To represent the resulting extent of oxidation, the thickness of the oxide film—if the oxidation product were present as a film only, with no dissolved oxygen—was calculated. Considering a hemispherical melt pool, the amount of W oxide in ppm is calculated using Eq. 2.

2 $${\text{Oxide}}\left( {{\text{ppm}}} \right) = \frac{{8\text{L}\rho_{{{\text{WO}}_{2} }} }}{{\pi \text{W}\rho_\text{W} }} \times 10^{6}$$

where L is the thickness of the oxide film, a function of experimental meltpool width, given by Eq. 3.

3 $$\text{L }\left(\text{m}\right)= \frac{{\text{mP}}_{\text{O}_2}{\text{WM}}_{{\text{WO}}_{2}}}{\text{RV}{\rho }_{{\text{WO}}_{2}}{\text{T}}_{\text{film}}}$$

m is the mass transfer coefficient, given by Eq. 4:

4 $$\text{m}= \frac{2\text{S}_{\text{h}}\text{D}_\text{gas}}{\pi W}$$

D_gas_ is the diffusivity of oxygen in the gas—0.00049 m^2^/s.

S_h_ is the Sherwood number, given by Eq. 5:

5 $$\text{S}_\text{h}\approx 4.9442 \sqrt{\frac{\text{{W}{V}}_\text{gas}}{2 \pi {\text {D}}_\text{gas}}}$$

V_gas_ is the velocity of the gas—10 m/s. ρ_WO2_ is the density of WO_2_ oxide at room temperature—10.8 g/cc. ρ_W_ is the density of W oxide at room temperature—19.25 g/cc. P_O2_ is the partial pressure of oxygen in the build chamber—87.1 Pa. M_WO__2_ is the molar mass of WO_2_—0.21584 kg. R is the universal gas constant. T_film_ is the temperature of oxide film, that is assumed as an average of melting point and room temperature—1990.5 K. V is the laser scan speed. W is the meltpool width obtained from the cross-section images of the single tracks in the Trumpf PBF-LB system.

An average of three meltpool width values were considered for the Trumpf PBF-LB system for these calculations.

#### Characterization

Top-down micrographs of the single tracks and cross-sectional images were taken using a Thermo Fisher Phenom ParticleX scanning electron microscope (SEM); top-down images were obtained in secondary electron (SE) mode, and melt pool cross-sectional images were obtained in backscatter (BSE) mode. An accelerating voltage of 20 kV and a working distance of 8–9 mm were used. To reveal the microstructures in cross-sections W, the samples were mechanically polished using the procedure in^37^ and etched for 10–15 s by swabbing with an etchant containing 0.2 g KOH, 1.5 g of K_3_[Fe(CN)_6_] and 10 ml distilled water. Additionally, the procedure according to ASTM E112 was used to measure the grain size of the melt pool cross-sections and the plan-view (top-down) images^38^. The intercept length was measured on the cross-sections between 0° and 180° rotation with a step size of 25°, similar to the methodology shown in^37^. For the plan views, two different images per sample were used with a total of 20 intercept measurements. The aspect ratio, defined as the ratio of grain length to width, was calculated for a total of 20 measurements.

To reveal the bubble and notch formation in the synchrotron X-ray radiography, the methodology similar to Martin *et. al* was implemented^39^. Additionally, in order to understand the spatter behavior, the frame difference approach was performed on the image stack to reveal spatter, following which manual particle tracking in Fiji is used to track particles^40^.

## Electronic Supplementary Material

Below is the link to the electronic supplementary material.

Supplementary Material 1

## Data Availability

The raw and processed data are available upon reasonable request from the corresponding author, Bryan A. Webler.
